# Editorial: The impact of the DNA damage response on anti-tumor immunity

**DOI:** 10.3389/fimmu.2022.1026194

**Published:** 2022-09-27

**Authors:** Gang Chen, Shengtao Zhou, Chaoyang Sun, Leng Han

**Affiliations:** ^1^ National Clinical Research Center for Gynecology and Obstetrics, Tongji Hospital, Tongji Medical College, Huazhong University of Science and Technology, Wuhan, China; ^2^ West China Second University Hospital, Sichuan University, Chengdu, China; ^3^ Center for Epigenetics and Disease Prevention, Institute of Biosciences and Technology, Texas A&M University, Houston, TX, United States

**Keywords:** DNA damage response, onco-immunology, DDR-targeting therapy, Immunotherapy, Immune checkpoint blockade

This editorial summarizes the contributions to the Research Topic “Impact of DNA Damage Response on Anti-tumor Immunity” that appears in the journal *Frontiers in Immunology*.

Drugs targeting DNA damage repair (DDR) are being applied in an increasing number of anti-tumor therapies. Preclinical studies have shown that in addition to their direct toxic effect on cancer cells, DDR-targeted drugs are also associated with increased levels of tumor-infiltrating lymphocytes, greater genomic instability, and higher tumor mutational burden (TMB) in cancer, enhancing the antitumor immune effect. Exploiting recent advancements in the mechanisms of immunological change during DDR-targeted therapy and in the efficacy of DDR inhibitors in combination with immune checkpoint inhibitors (ICIs) has already led to a significant leap forward in anti-tumor therapies. It is therefore of great interest to continue working to understand the consequences of DDR-associated therapies in patients and to identify novel DDR targets and combination strategies, as this will ultimately benefit clinical practice.

This Research Topic explored recent advancements in relation to DDR, with a focus on 1) understanding the relationship between DDR-targeted therapies and patients’ response to immune checkpoint blockade (ICB) agents, as well as the immune-activating properties of DDR-targeted therapies; 2) the role of DNA repair defects (including DDR gene mutations, genomic instability, and the expression profiles of DDR genes) in predicting immunotherapy response; 3) the mechanisms of chemotherapy, radiation, and DDR-targeted therapy–induced immunogenicity, including the cytosolic DNA–sensing cGAS/STING-IFN pathway, neoantigen, or tumor-associated antigen; 4) spatiotemporal and sequential treatment strategies for patients who are candidates for combination therapies with DDR inhibitors and immune therapy; 5) the identification of novel DDR targets and combination strategies; 6) providing a clinical update on the combination of DDR inhibitor and immunotherapy results and offering future perspectives.

In one study, Zhou et al. suggested that the activation level of the DDR pathway may be a novel predictive marker for immunotherapy efficacy in patients with metastatic urothelial carcinoma (mUC). In this study, a published immunotherapy cohort with genome, transcriptome, and survival data for 348 mUC patients was used and validated by an external cohort (The Cancer Genome Atlas Bladder Cancer) and the GSE78220 cohort. The results showed that the DDR enrichment score-high (DSSH) group was associated with longer overall survival times and higher TMB, neoantigen load, immune-related gene expression levels, and immune-activated cell patterns (increased activated memory CD4 T cells, activated natural killer [NK] cells, and M1 macrophages, but a lower proportion of monocytes). Further investigation of the mechanism indicated that the higher immune activation state may be correlated with a down-regulation in the transforming growth factor β receptor signaling pathway.

A second study by Lou et al. presented a novel patient-level DDR pathway profiling approach that revealed distinct DDR pathway clusters using a total of 2,019 gastric cancer (GC) samples from Harbin Medical University Cancer Hospital in China and 12 public data sets. This study demonstrated a clear distinction in the DDR pathway profiling between tumor and normal tissues and also revealed patient-level variations, which may contribute to explaining the high heterogeneity of human GC in terms of biological features and treatment outcomes. With additional prospective validation in clinical and mechanistic studies, the identified DDR pathway signature could become a powerful tool for stratifying advanced-stage GC patients toward personalized treatments incorporating chemotherapy and immunotherapy.

Another study by Xiong et al. focused on co-mutations (co-mut+) in DDR pathways and explored the predictive role of co-mut+ status in the efficacy of ICIs in 853 non–small cell lung cancer (NSCLC) patients from the OAK and POPLAR trials. Their results showed that the interaction between co-mut status and treatment was significant for progression-free survival and overall survival. In patients with negative or low programmed death receptor-ligand 1 (PD-L1) expression, co-mut+ status still predicted improved clinical outcomes with atezolizumab therapy, suggesting that co-mut status may be a promising predictor of ICI therapy in NSCLC.

By detecting micronuclei or cytoplasmic chromatin fragments induced by DNA damage, the cGAS/STING pathway can mediate the interplay between cytotoxic effects and immune stimulation, exerting a dichotomous effect on tumor tissue. Gan et al. discussed the biological mechanisms of the cGAS/STING pathway, its dichotomous role in tumors, and the latest advances with respect to STING agonists and antagonists. They concluded that exploring the molecular details of the cGAS/STING pathway may enhance the current understanding of the antitumor mechanism of innate immunity and may provide a theoretical basis for future drug design for the treatment of tumors. The co-administration of classical therapies and STING agonists has demonstrated an excellent synergistic antitumor effect in several preclinical tumor models. Therefore, the comparison of the effects of various combination therapies and the exploration of their mechanisms is of utmost significance.

Xie et al. reviewed the rationale and clinical applications of targeting the DNA repair response to promote immunotherapy in ovarian cancer. In this review, the authors provided an overview of the interconnectivity between DDR pathway deficiency and the immune response, summarized available clinical trials on combination therapy in ovarian cancer, discussed the potential predictive biomarkers that can be utilized to guide the use of combination therapy, ultimately concluded that the combination of DDR inhibitors with ICI represents an attractive therapeutic strategy, with the potential to improve the clinical outcomes of patients with ovarian cancer.

## Author contributions

GC and CS were guest associate editors of the Research Topic and wrote the paper text. SZ and LH were guest associate editors of the Research Topic and edited the text. All authors contributed to the article and approved the submitted version.

## Acknowledgments

We thank the authors of the papers published in this Research Topic for their valuable contributions and the referees for their rigorous review. We also thank the editorial board of *Frontiers in Immunology* for their support.

## Conflict of interest

The authors declare that the research was conducted in the absence of any commercial or financial relationships that could be construed as a potential conflict of interest.

## Publisher’s note

All claims expressed in this article are solely those of the authors and do not necessarily represent those of their affiliated organizations, or those of the publisher, the editors and the reviewers. Any product that may be evaluated in this article, or claim that may be made by its manufacturer, is not guaranteed or endorsed by the publisher.

